# Sex Differences in Comorbidities of Pediatric Craniosynostosis at Presentation

**DOI:** 10.1159/000528745

**Published:** 2022-12-21

**Authors:** Peyton Presto, Reagan A. Collins, John Garza, Omar Fadi Zeitouni, Laszlo Nagy

**Affiliations:** ^a^School of Medicine, Texas Tech University Health Sciences Center, Lubbock, Texas, USA; ^b^Department of Mathematics, The University of Texas Permian Basin, Odessa, Texas, USA

**Keywords:** Craniosynostosis, Pediatric craniosynostosis, Comorbidity, Sex differences, Neurosurgery

## Abstract

**Introduction:**

Craniosynostosis is a common pediatric presentation in which the premature fusion of one or more cranial sutures results in a misshapen skull. This birth defect is often associated with comorbidities due to structural impacts on nearby anatomical features. While there is some evidence for a male predominance among craniosynostosis patients, little has been investigated regarding sex differences in comorbidities of this condition. This study seeks to explore potential sexual dimorphisms in craniosynostosis patients at the time of presentation.

**Methods:**

We conducted a retrospective, cross-sectional review of male and female non-syndromic craniosynostosis (NSC) patients between the ages of 1 month and 9 years that were evaluated at a 500-bed academic hospital or a 977-bed private hospital in Lubbock, TX, USA. Common comorbidities including ophthalmologic diagnoses, developmental delays, obstructive sleep apnea, chronic otitis media, hearing loss, chronic headaches, and seizure disorders were evaluated. The NSC cohort was compared to a similarly aged trauma group that represented the normal population.

**Results:**

175 NSC patients fit the inclusion criteria, of which 109 (62%) were male. A diagnosis of craniosynostosis was significantly associated with ophthalmological diagnoses (*p* < 0.0001), chronic otitis media (*p* < 0.0001), developmental delays (*p* < 0.0001), and hearing loss (*p* = 0.0047). Male NSC patients were less likely to present with ophthalmological diagnoses (*p* = 0.0010) or hearing loss (*p* = 0.0052) than females.

**Conclusions:**

Our findings expand on current literature evaluating possible comorbidities of NSC, particularly supporting the association with ophthalmological diagnoses, chronic otitis media, developmental delays, and hearing loss. We also report sex differences in ophthalmological diagnoses and hearing loss for NSC patients. These findings can serve to educate physicians of symptoms requiring prompt recognition and management in these patients.

## Introduction

Craniosynostosis is defined as the premature fusion of one or more cranial sutures and is estimated to appear in 1 in 2,000–2,500 births [1, 2, 3, 4]. The metopic suture often closes at 6–12 months of age, while the other cranial sutures may remain open into adulthood. Craniosynostosis is classified as syndromic or non-syndromic, with the non-syndromic type typically involving a single suture [3]. Syndromic craniosynostosis is often linked to genetic disorders such as Apert, Crouzon, or Pfeiffer syndromes, whereas non-syndromic craniosynostosis (NSC) develops as an isolated disorder [3].

Following the premature fusion of the cranial sutures, the infant sustains no growth in the affected area of the cranium while the non-fused regions of the skull continue to grow, ultimately resulting in cranial deformation, compromised brain growth, and increased intracranial pressure (ICP) [1, 5]. With these structural impacts on nearby anatomical features and the subsequent risk of increased ICP, craniosynostosis patients may present with consequent ophthalmological diagnoses, such as strabismus, papilledema, or exophthalmos [6, 7, 8, 9, 10]. Associations have also been reported with developmental delays, obstructive sleep apnea, chronic otitis media, hearing loss, chronic headaches, and seizure disorders [11, 12, 13, 14, 15, 16, 17, 18, 19, 20, 21]. Despite these correlations, substantial evolution in NSC management has led to improved outcomes in these patients, and the genetic pathophysiology of NSC craniosynostosis is becoming increasingly better understood with the development of next-generation sequencing technologies [22, 23]. This could suggest that future NSC treatments may be guided by illuminating the underlying genetic causes of these associated conditions, potentially leading to nonsurgical therapeutic options [24]. Additionally, as there is some lack of consensus among surgeons regarding the best NSC management practices [25], treatment protocols may instead develop a stronger focus on management of associated symptoms. Therefore, determination of risk factors for NSC comorbidities, including potential sex-specific differences, is an important area of future investigation.

Studies have shown a male-to-female ratio of 2:1 in overall incidence of craniosynostosis, with males accounting for a majority of metopic, sagittal, and lambdoid synostosis cases [26]. Conversely, a female predominance was observed in coronal synostosis [26, 27]. Despite these reports, little has been explored with regard to sex differences in comorbidities of the diagnosis, particularly related to the patient's age at presentation [26]. The limited sample size in most NSC clinical studies likely precludes analyses by sex, though this omission may create misinformation regarding the burden of NSC comorbidities and hinder prompt management of these conditions. We aimed to assess current incidences of pediatric craniosynostosis comorbidities at presentation, specifically regarding potential sexual dimorphisms among the diagnoses.

## Material and Methods

### Patient Selection

This retrospective study received Institutional Review Board (IRB) approval at Texas Tech University Health Sciences Center (TTUHSC) with a waiver of the informed consent requirement. Following IRB approval, we isolated a group of 417 patients who were diagnosed as having an anomaly of skull and face bones at either University Medical Center (UMC) or Covenant Medical Center (CMC) in Lubbock, TX, USA, between January 2011 and December 2018. Anomalies of skull and face bones were identified by International Classification of Diseases (ICD)-9 (756.0) and ICD-10 (Q75.0) codes. From these cases, patients with a diagnosis of NSC between the ages of 1 month to 9 years were selected for our initial cohort. Patients were excluded if they received a primary diagnosis other than NSC (such as syndromic or complex craniosynostosis including Apert, Crouzon, Muenke, Pfeiffer, and Saethre-Chotzen syndromes, congenital absence of cranial bones, etc.) or if they fell outside the specified age range at time of diagnosis. As we were interested in examining the concurrence of ophthalmological findings, which are known to be a common craniosynostosis comorbidity, patients were also excluded if they had not received an ophthalmological exam. Our final patient cohort was comprised of 175 NSC patients (109 males and 66 females).

### Data Collection

The electronic medical record (EMR) for each patient was reviewed for demographic as well as diagnostic and therapeutic information. The subtype of NSC (i.e., sagittal, coronal, metopic, or pansynostosis) was recorded. Ophthalmological diagnoses were obtained from clinic notes and included exophthalmos, strabismus, blindness, amblyopia, congenital malformation, retinopathy of prematurity, papilledema, nystagmus, and unspecified visual disturbances. Information regarding other common comorbidities of craniosynostosis (chronic otitis media, developmental delays, hearing loss, obstructive sleep apnea, chronic headaches, and seizure disorders) was obtained from EMR documentation available at the time of initial NSC presentation. Patients were considered to have chronic otitis media if they exhibited one or more of the following criteria: (1) recurrent middle ear infections resulting in repeated (>1) clinic visits over a 6-month period; (2) perforation of the tympanic membrane accompanied by a middle ear infection; or (3) history of tympanostomy tube placement. Developmental delays in patients were identified by the following ICD codes: (1) ICD-9 (315) and ICD-10 (F80-89), corresponding to pervasive and specific developmental disorders of speech/language, scholastic skills, or motor function, as well as unspecified disorders of psychological development; or (2) ICD-9 (783.4) and ICD-10 (R62.0-5), corresponding to delayed milestones or lack of expected normal physiological development in childhood. Patients were considered to have hearing loss if they exhibited >25 dB conductive, sensorineural, or mixed hearing loss on audiogram. Patients were considered to have obstructive sleep apnea if they exhibited an apnea-hypoxia index >1 or a minimum oxygen saturation of <92% on polysomnogram. Patients with chronic headaches were identified by the following ICD codes: (1) ICD-9 (346.7-9) and ICD-10 (G43.7-9), corresponding to chronic migraines; (2) ICD-9 (339.12) and ICD-10 (G44.22), corresponding to chronic tension-type headaches; and (3) ICD-9 (339.42) and ICD-10 (G44.52), corresponding to new persistent daily headaches. Finally, patients were considered to have a seizure disorder if they had prior documentation of >2 unprovoked seizures occurring more than 24 h apart.

### Trauma Control Group

The presence of ophthalmological diagnoses and other known craniosynostosis comorbidities (see Data Collection) was collected for a group of 249 similarly aged patients (124 males and 125 females) seen in the UMC or CMC emergency departments over the same time period. These patients were initially assessed for trauma but found to have no trauma-induced skull anatomical deformities on neuroimaging. Patients were excluded from this cohort if they had received any prior neurosurgical intervention or had received a diagnosis of any preexisting neurologic or cranial structural anomaly (including craniosynostosis), or if no ophthalmological documentation was available in the EMR. This cohort served as a representation of the normal population and was used as a control group for the purposes of this study.

### Statistical Analysis

The project is a retrospective, observational, and cross-sectional study using descriptive and inferential statistics. Statistical analyses used independent samples, two-sided *p* values, and a significance level of α = 0.05. Continuous variables are summarized using the mean and standard deviation with differences evaluated using the unequal variance *t* test. Categorical variables are summarized using counts and percentages with differences evaluated using Fisher's test. Power analyses for Fisher's test and the *t* test corresponding to the sample sizes of the project are provided in online supplementary Figure 1 (for all online suppl. material, see www.karger.com/doi/10.1159/000528745). Standardized effect sizes are reported as the standardized mean difference (SMD). No adjustments to *p* values for multiple comparisons have been made since the study is intended to evaluate the plausibility of differences. Multivariate differences between patient groups were evaluated using the multivariate Welch *t* test [28] based on the adjusted Bray-Curtis dissimilarity with a single *p* value computed using 10,000 random permutations [29]. Subgroup analyses by age were performed to review the consistency of associations. Statistical analyses were completed using R version 4.1.1 and RStudio version 1.4.1717.

## Results

### Patient Demographics

175 NSC patients fit the inclusion criteria and were compared to 249 trauma patients. Of the 175 NSC patients, 109 (62%) were male and 66 (38%) were female with a mean (standard deviation) age of 14.3 (13.5) months. Table 1 summarizes characteristics and outcomes for trauma and NSC patients (effect sizes reported as odds ratios in online suppl. Table 1). As there were no significant associations between NSC subtype and any of the investigated outcomes (online suppl. Table 2), all NSC patient subtypes were combined into a single NSC group. In the entire cohort of NSC patients, almost a quarter (24%) had also received a diagnosis of chronic otitis media. Similarly, one-third of NSC patients were also found to have ophthalmological diagnoses (33%) or developmental delays (34%). Smaller proportions of NSC patients presented with hearing loss (11%), chronic headaches (12%), obstructive sleep apnea (9%), or seizure disorders (14%). Comparatively, almost 20% of the trauma control group had received a chronic headache diagnosis, though the prevalence of all other comorbidities was found to be lower than in the NSC cohort (chronic otitis media, 2%; ophthalmological diagnoses, 10%; developmental delays, 11%; hearing loss, 4%; obstructive sleep apnea, 4%; seizure disorders, 13%).

**Fig. 1:**
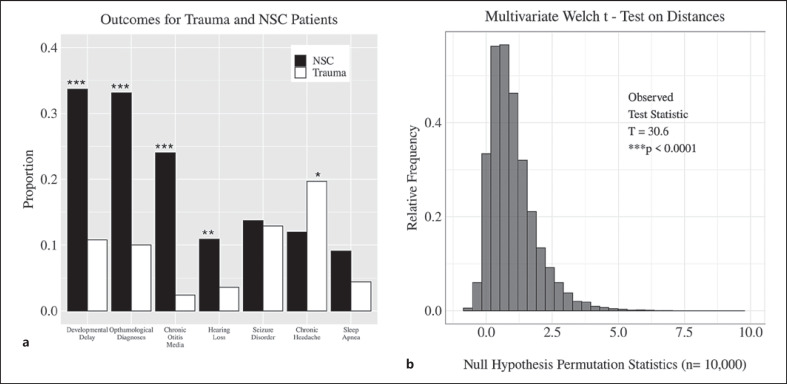
Comorbidity outcomes in NSC and trauma patients. **a** Comparison of proportions of comorbidities at presentation among NSC (*n* = 175) and trauma (*n* = 249) patients. A significantly higher proportion of NSC patients presented with developmental delays, ophthalmological diagnoses, chronic otitis media, and hearing loss compared to patients in the trauma control group. Fisher's test, *p < 0.05, **p < 0.01, ***p < 0.0001. **b** Multivariate Welch *t* test showing the significant difference in prevalence of comorbidities among the groups, ***p < 0.0001.

Compared to trauma patients, NSC patients were younger (SMD = 1.3377, *p* < 0.0001) and more likely to be male (SMD = 0.2536, *p* = 0.0131). A diagnosis of NSC was significantly associated with ophthalmological diagnoses (SMD = 0.5850, *p* < 0.0001), chronic otitis media (SMD = 0.6729, *p* < 0.0001), developmental delays (SMD = 0.5716, *p* < 0.0001), and hearing loss (SMD = 0.2823, *p* = 0.0047) when compared to patients in the trauma control group (Fig. 1a). However, trauma patients were more likely to present with chronic headaches than NSC patients (SMD = 0.2115, *p* = 0.0458) (Fig. 1a). The multivariate Welch *t* test based on adjusted Bray-Curtis dissimilarity confirmed a significant difference (*p* < 0.0001) in outcomes between trauma and NSC patients (Fig. 1b).

### Sex Differences

Table 2 summarizes characteristics and outcomes for male and female NSC patients (effect sizes reported as odds ratios in online suppl. Table 3). The most common NSC subtype for both male and female patients was sagittal synostosis, accounting for 56% of female and 56% of male primary diagnoses. The next most common subtypes were metopic synostosis (female, 35%; male, 23%), coronal synostosis (female, 8%; male, 18%), and pansynostosis (female, 2%; male, 3%), respectively. As there were no significant differences between the sexes with regard to NSC patient subtypes, all NSC primary diagnoses were combined into singular NSC female and NSC male groups. With regard to associated comorbidities, almost one-half of female NSC patients (49%) presented with at least one ophthalmological diagnosis; in comparison, only 24% of males were found to have an ophthalmological comorbidity. Similarly, 20% of female NSC patients presented with hearing loss, whereas only 6% of male NSC patients shared this comorbidity. Despite the significant prevalence of developmental delays and chronic otitis media in our combined NSC patient cohort (see Patient Demographics), no sex differences were observed for either condition (developmental delay − female, 38%, and male, 31%; chronic otitis media − female, 24%, and male, 24%). Female and male NSC patients showed no statistically significant differences in the prevalence of sleep apnea (female, 15%; male, 6%), seizure disorders (female, 9%; male, 17%), or chronic headaches (female, 14%; male, 11%).

**Fig. 2:**
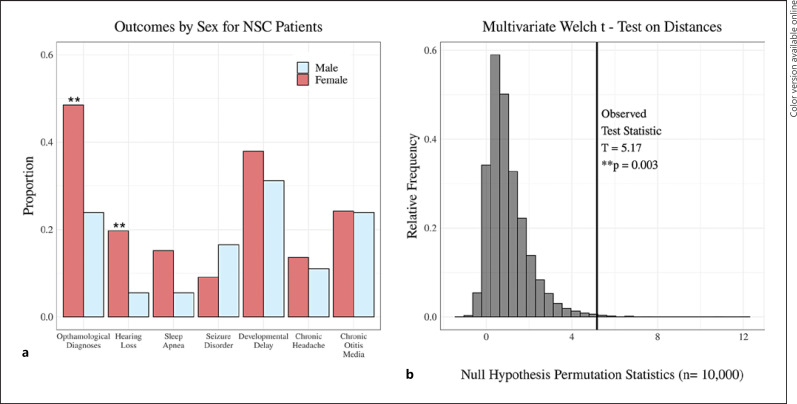
Comorbidity outcomes in NSC patients stratified by sex. **a** Comparison of proportions of comorbidities at presentation among male (*n* = 109) and female (*n* = 66) NSC patients. A significantly higher proportion of female NSC patients presented with ophthalmological diagnoses and hearing loss compared to male NSC patients. Fisher's test, **p < 0.01. **b** Multivariate Welch *t* test showing the significant difference in prevalence of comorbidities among males and female NSC patients, **p = 0.003.

Male and female NSC patients had similar mean age (SMD = 0.1333, *p* = 0.3940). Male patients were less likely to present with ophthalmological diagnoses (SMD = 0.5304, *p* = 0.0010) or hearing loss (SMD = 0.4378, *p* = 0.0052) than females (Fig. 2a). The multivariate Welch *t* test based on adjusted Bray-Curtis dissimilarity confirmed a significant difference (*p* = 0.0030) in outcomes between male and female patients (Fig. 2b). Further subgroup analysis by age demonstrated consistently lower rates in males for ophthalmological diagnoses and hearing loss regardless of age at initial presentation (Tables 3 and 4; effect sizes presented as odds ratios in online suppl. Tables 4 and 5). Importantly, no significant differences were found between male and female trauma patients for any outcome (online suppl. Table 6), suggesting that the observed sex differences among NSC patient comorbidities may be attributed to sex-specific mechanisms in NSC as opposed to inherent baseline differences in male and female prevalence rates.

## Discussion

Craniosynostosis cases are most commonly non-syndromic, affecting only a single suture [30, 31]. Comorbidities of NSC have been widely reviewed, with several groups providing evidence that children with this condition are at an increased risk for persistent neuropsychological deficits [32, 33, 34, 35]. Though often attributed to an increase in ICP, especially among syndromic cases [36], many comorbid conditions may arise due to structural impacts of brain growth on neighboring anatomical features [37]. Additionally, many studies have documented an association between NSC and various neuropsychological deficits, but little has been described with regard to sex differences among comorbid conditions. To our knowledge, we provide the first evidence for sexually dimorphic NSC comorbidities. While an overall NSC diagnosis was significantly associated with ophthalmological diagnoses, chronic otitis media, hearing loss, and developmental delays (see Fig. 1; Table 1), further analysis by sex revealed that male patients were less likely to present with ophthalmological diagnoses or hearing loss than female patients (see Fig. 2; Table 2).

Ophthalmological diagnoses among NSC patients are well documented in the literature [6, 7, 8, 9, 10]. Despite wide variation across studies, a recent review by Rostamzad et al. [38] reported that the overall prevalence rates of functional ophthalmic abnormalities in this group may range from 19 to 35%. While not further broken down by sex, this is in alignment with our reported finding of ophthalmological diagnoses in 33% of combined male and female NSC cases. Importantly, however, we found a prevalence rate among female NSC patients as high as 49%; this suggests that females with NSC may be more likely to suffer from ophthalmic abnormalities than their male counterparts. To the best of our knowledge, this potential female predominance has not been previously reported. Though visual anomalies may be less severe than those of syndromic cases [6], ophthalmologic evaluation in NSC cases remains essential to minimize the risk of vision loss that accompanies more critical ophthalmological dysfunctions such as papilledema and amblyopia. It is important to note the general consensus that surgical intervention in NSC patients before 1 year of age is preferred to minimize harmful effects on brain development and to avoid more invasive procedures at a later timepoint [6, 39]. While our findings did not reveal an increased association of ophthalmological diagnoses among NSC patients with age (see Table 3), there is evidence that deficits in visuomotor function may affect a child's development in other domains [40]. Wallace et al. [41] reported that in a large cohort of school-age children, children with NSC were more likely to experience deficits in manual dexterity than in visual processing when compared to the non-NSC cohort. Our findings support the current recommendations for early ophthalmologic exams in NSC patients to address both direct visual deficits and further implications on other neurocognitive domains, especially among female patients.

The abnormal skull growth associated with craniosynostosis may impede development of the middle ear, increasing the risk of audiologic deficits such as chronic otitis media and hearing loss [17]. Though these outcomes are more commonly associated with syndromic cases [17, 19, 42, 43], some studies have explored the presence of chronic otitis media and hearing loss in the NSC population. Grewal et al. [18] reported that 36% of their 113-patient NSC cohort had experienced at least one episode of otitis media, falling within the upper limit of normal when compared to historical rates among normocephalic children, and only 9% of NSC patients were found to have hearing loss; however, this study did not report a breakdown of prevalence rates by sex. Similarly, Prager et al. [20] found 26% of 57 NSC patients presented with hearing loss, a frequency that was not significantly higher than the normal population of a similar age, and only a quarter of NSC patients with hearing loss were female. We report lower rates of chronic otitis media and hearing loss in our NSC cohort (24% and 11%, respectively) but still found these to be significantly higher than those of control patients. We also found a significantly higher prevalence of hearing loss among female NSC patients when compared to their male counterparts (20% compared to 6%, respectively). While we did not find evidence for an increased association of hearing loss among NSC patients with age (see Table 4), some studies have reported that untreated otitis media may cause audiological deficits in syndromic craniosynostosis [17, 44, 45, 46]. Thus, the rates of chronic otitis media and hearing loss in our NSC patient cohort compared to the control group suggest the importance of early audiologic evaluation even among NSC cases.

Reports of developmental delays in NSC patients are conflicting in the literature. Some studies suggest that the learning and/or intellectual disabilities were more prevalent in NSC patients than in the general population, with rates of developmental delays ranging from 35–80% of children with NSC [13, 33, 47]. However, others report a normal distribution of full-scale IQ scores in a NSC cohort [14]. These conflicting findings may be explained by differences within the ages of NSC patients at evaluation, as many learning disabilities are not diagnosed until children are observed to have difficulties in school. Studies that focus on NSC during infancy may overlook cognitive deficits that become more apparent at a later age. There is evidence that as age of evaluation increases, the percentage of NSC patients that present with speech-language, cognitive, and behavioral abnormalities also increases [48, 49]. A strength of the current study is our large NSC cohort that spans patients from infancy to school age. As we observed a significantly higher rate of developmental delays in the NSC group (34% compared to 11% of our trauma control group), we recommend longitudinal assessments for learning disabilities and cognitive deficits in the care for these patients.

The increased prevalence of NSC in males is well documented, with many studies reporting a 2:1 male:female ratio [11, 14, 26, 27, 50]. We report similar findings, as 62% of our NSC patients were male. However, our data differed from prior studies that reported a female predominance of coronal synostosis prevalence [26, 27], as we found that 18.1% of male but only 7.6% of female NSC patients fell into this subtype (see Table 2). Preclinical studies have explored the well-established male predominance of NSC, offering explanations ranging from the role of hormonal receptors on bone formation to transcriptional effects of sex on osteoblast differentiation [51, 52, 53]. Despite these efforts, a complete understanding of the male predominance of NSC has yet to emerge, and even less has been investigated regarding sexually dimorphic NSC presentations. This omission may be attributed to the small sample size of NSC patients in most studies, which prevents further analysis by sex. While one study reported that males with NSC were more likely to experience learning disabilities than females with NSC (50% vs. 30%, respectively) [54], we did not find an increased association of developmental delays among males in our NSC cohort. Our findings instead suggest sex differences in ophthalmologic and audiologic comorbidities, as NSC males were less likely to present with ophthalmological diagnoses or hearing loss than NSC females across all age groups. Early diagnosis and management of both of these conditions are essential to minimizing the risk for permanent damage [7, 8, 9, 10, 19]. Therefore, we recommend close monitoring of NSC patients to ensure any yet unreported sequelae associated with NSC are promptly managed.

Our findings highlight the burden of NSC comorbidity, even at diagnosis. When compared to an age-matched control population, almost all evaluated comorbidities were more prevalent in the NSC cohort. The highest rates were ophthalmological diagnoses, developmental delays, and chronic otitis media, all of which were 20% more prevalent among NSC patients. The observed sex-specific differences in the NSC comorbidity burden, particularly the fact that almost one-half of female NSC patients suffered from ophthalmic abnormalities at presentation, indicate the urgent need to bring clinical attention to this issue. As these sexual dimorphisms were not present in our age-matched control population, further investigation with regard to sex-specific mechanisms of NSC is warranted. Our findings provide support for the inclusion of both sexes, as well as the specification of sex among comorbid conditions, in future NSC studies. Sex-specific clinical profiles of NSC may be critical considerations in future NSC management protocols, particularly as the field gains increasing knowledge of the genetics underlying NSC.

Limitations include the retrospective nature of the study. Additionally, since our exclusion criteria required the documentation of an ophthalmological exam, there is potential for selection bias that may contribute to the distribution of outcomes in our NSC cohort. Due to the limited sample size of each reported ophthalmological diagnosis, no further analysis could be conducted based on ophthalmological finding subtypes. Though we elected to combine NSC subtypes into a single cohort due to inadequate sample sizes, future studies may expand on outcomes based on NSC subtype to investigate potential sex-specific associations.

## Conclusions

Pediatric craniosynostosis can lead to increased ICP, structural impacts on brain growth, and associated sequela. Prompt diagnosis and management is essential to prevent any long-term consequences for these patients. Our findings expand on current literature to suggest potential sex differences in craniosynostosis comorbidities and serve to educate physicians of symptoms that require recognition and early management in these patients.

## Statement of Ethics

This study protocol was reviewed and approved by the Institutional Review Board for the Protection of Human Subjects at Texas Tech University Health Sciences Center in Lubbock, TX, USA, approval number L19-075, with a waiver of the informed consent requirement.

## Conflict of Interest Statement

The authors have no conflicts of interest to declare.

## Funding Sources

This research did not receive any funding from agencies in the public, commercial, or not-for-profit sectors.

## Author Contributions

Peyton Presto: conceptualization, methodology, investigation, and writing − original draft. Reagan A Collins: conceptualization, methodology, and writing − original draft. John Garza: formal analysis, visualization, and writing − review and editing. Omar Fadi Zeitouni: conceptualization and methodology. Laszlo Nagy: conceptualization, methodology, supervision, writing − review and editing, and project administration.

## Data Availability Statement

All data generated or analyzed during this study are included in this article and its online supplementary material. Further inquiries can be directed to the corresponding author.

## Supplementary Material

Supplementary data

## Figures and Tables

**Table 1:** Characteristics and outcomes for NSC and trauma patients

	NSC (*n* = 175)	Trauma (*n* = 249)	SMD	*p* value
Characteristics				
Age, months	14.3 (13.5)	35.6 (17.8)	1.3377	<0.0001
Sex, females	66 (37.7)	125 (50.2)	0.2536	0.0131
Outcomes				
Chronic otitis media	42 (24.0)	6 (2.4)	0.6729	<0.0001
Ophthalmological diagnoses	58 (33.1)	25 (10.0)	0.5850	<0.0001
Developmental delay	59 (33.7)	27 (10.8)	0.5716	<0.0001
Hearing loss	19 (10.9)	9 (3.6)	0.2823	0.0047
Chronic headache	21 (12.0)	49 (19.7)	0.2115	0.0458
Sleep apnea	16 (9.1)	11 (4.4)	0.1888	0.0677
Seizure disorder	24 (13.7)	32 (12.9)	0.0254	0.8843

Patient demographics revealed that NSC (*n* = 175) patients were younger and more likely to be male than the trauma control (*n* = 249) patients; unequal variance *t* test. A significantly higher proportion of NSC patients presented with chronic otitis media, ophthalmological diagnoses, developmental delay, and hearing loss compared to patients in the trauma control group, while trauma patients were more likely to present with chronic headaches than NSC patients; Fisher's test. SMD, standardized mean difference.

**Table 2:** Characteristics and outcomes of male and female NSC patients

	Female (*n* = 66)	Male (*n* = 109)	SMD	*p* value
Characteristic				
Age, months	13.2 (11.8)	14.9 (14.5)	0.1333	0.3940
Primary diagnosis				
Coronal synostosis	5/66 (7.6)	20/109 (18.3)	0.3895	0.1190
Metopic synostosis	23/66 (34.8)	25/109 (22.9)		
Pansynostosis	1/66 (1.5)	3/109 (2.8)		
Sagittal synostosis	37/66 (56.1)	61/109 (56.0)		
Outcome				
Ophthalmological diagnoses	32/66 (48.5)	26/109 (23.9)	0.5304	0.0010
Hearing loss	13/66 (19.7)	6/109 (5.5)	0.4378	0.0052
Sleep apnea	10/66 (15.2)	6/109 (5.5)	0.3211	0.0549
Seizure disorder	6/66 (9.1)	18/109 (16.5)	0.2235	0.1830
Developmental delay	25/66 (37.9)	34/109 (31.2)	0.1410	0.4108
Chronic headache	9/66 (13.6)	12/109 (11.0)	0.0800	0.6362
Chronic otitis media	16/66 (24.2)	26/109 (23.9)	0.0091	1.0000

Patient demographics revealed no significant age difference between male (*n* = 109) and female (*n* = 66) NSC patients; unequal variance *t* test. No significant differences were observed between male and female NSC patients in primary NSC subtype diagnosis. A significantly higher proportion of female NSC patients presented with ophthalmological diagnoses and hearing loss compared to male NSC patients; Fisher's test. SMD, standardized mean difference.

**Table 3:** Subgroup analyses for the association of ophthalmological diagnoses with sex among NSC patients

Age, months	Outcome	Female	Male	SMD	*p* value
All data	Ophthalmological diagnoses	32/66 (48.5)	26/109 (23.9)	0.5304	0.0010
≤6	Ophthalmological diagnoses	8/23 (34.8)	8/42 (19.0)	0.3605	0.2287
6–12	Ophthalmological diagnoses	11/21 (52.4)	8/27 (29.6)	0.4755	0.1426
12–24	Ophthalmological diagnoses	9/14 (64.3)	5/17 (29.4)	0.7459	0.0759
≥24	Ophthalmological diagnoses	4/8 (50.0)	5/23 (21.7)	0.6166	0.1845

Female NSC patients (*n* = 66) consistently demonstrated higher rates of presentation with ophthalmological diagnoses than male NSC patients (*n* = 109); Fisher's test. SMD, standardized mean difference.

**Table 4:** Subgroup analyses for the association of hearing loss with sex among NSC patients

Age, months	Outcome	Female	Male	SMD	*p* value
All data	Hearing loss	13/66 (19.7)	6/109 (5.5)	0.4378	0.0052
≤6	Hearing loss	2/23 (8.7)	3/42 (7.1)	0.0575	1.0000
6–12	Hearing loss	6/21 (28.6)	2/27 (7.4)	0.5732	0.1146
12–24	Hearing loss	3/14 (21.4)	0/17 (0.0)	0.7385	0.0810
≥24	Hearing loss	2/8 (25.0)	1/23 (4.3)	0.6102	0.1557

Female NSC patients (*n* = 66) consistently demonstrated higher rates of presentation with hearing loss than male NSC patients (*n* = 109); Fisher's test. SMD, standardized mean difference.
