# Supplementary material for: Sex Differences in Comorbidities of Pediatric Craniosynostosis at Presentation
Source: Pediatr Neurosurg. 2022 Dec 21;58(1):8–17. doi: 10.1159/000528745 (PMC10064380; doi:10.1159/000528745)
Supplement: Supplementary file 1 — Supplementary data [file pne-0058-0008-s01.docx]

**Supplementary Figure 1.**

**
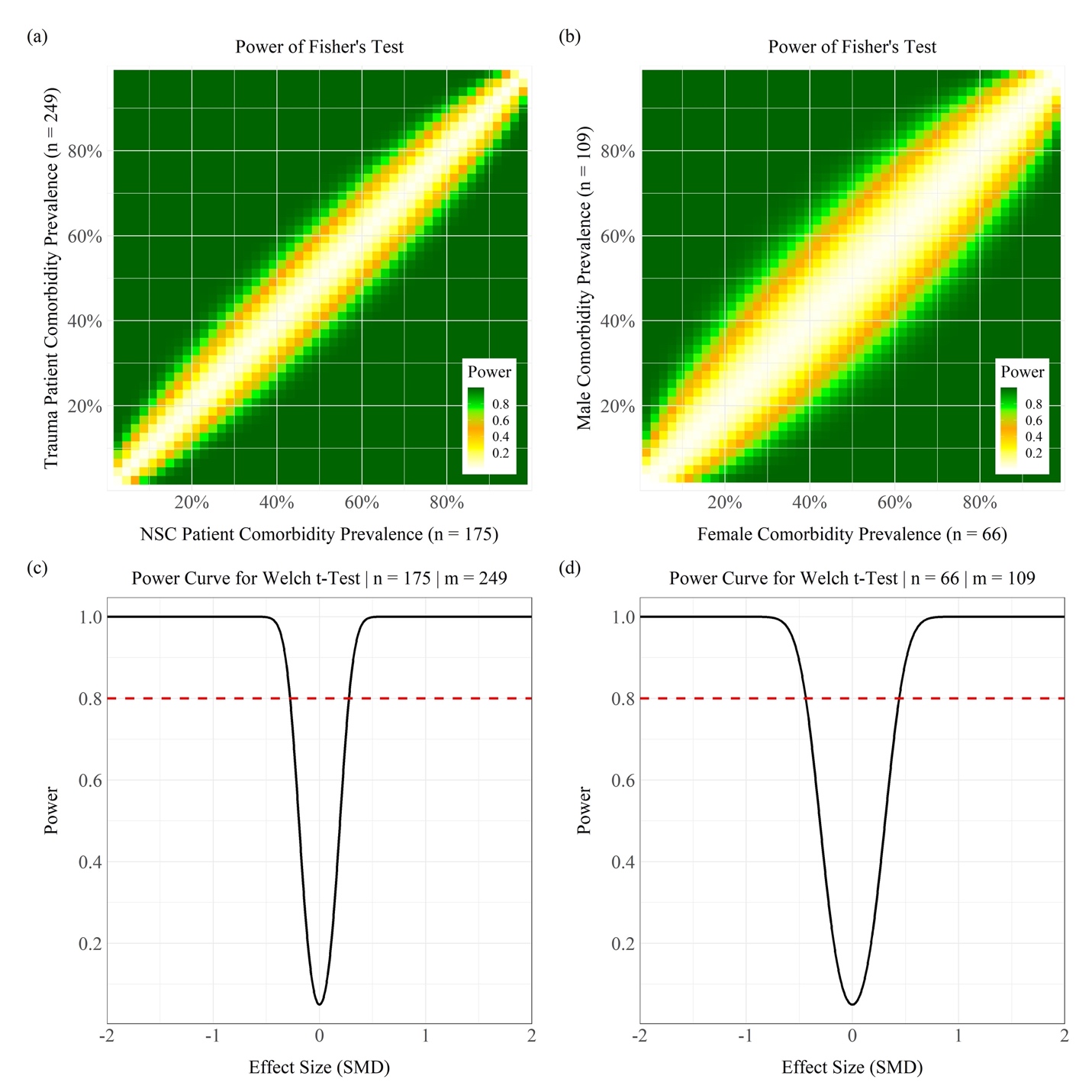
**

**Supplementary Figure 1:** Power analyses for Fisher’s test and the Welch t-test. **(a)** Power analysis for Fisher’s test of NSC (n = 175) and trauma control (n = 249) patient comorbidity prevalence at presentation. **(b)** Power analysis for Fisher’s test of male (n = 109) and female (n = 66) NSC patient comorbidity prevalence at presentation.  **(c)** Power curve for the Welch t-test of NSC (n = 175) and trauma control (n = 249) patient age at presentation. **(d)** Power curve for the Welch t-test of male (n = 109) and female (n = 66) NSC patient age at presentation.

| **Supplementary Table 1: Characteristics and outcomes of NSC and trauma patients using odds ratios** | | | | |
| --- | --- | --- | --- | --- |
| **Characteristic** | **NSC** | **Trauma** | **Odds Ratio** | **P Value** |
| Sex | 66/175 (37.7) | 125/249 (50.2) | 0.6014 | 0.0131 |
| **Outcome** |  |  |  |  |
| Ophthalmologic Diagnoses | 58/175 (33.1) | 25/249 (10.0) | 4.425 | <0.0001 |
| Chronic Otitis Media | 42/175 (24.0) | 6/249 (2.4) | 12.7204 | <0.0001 |
| Developmental Delay | 59/175 (33.7) | 27/249 (10.8) | 4.1669 | <0.0001 |
| Hearing Loss | 19/175 (10.9) | 9/249 (3.6) | 3.2387 | 0.0047 |
| Chronic Headache | 21/175 (12.0) | 49/249 (19.7) | 0.5573 | 0.0458 |
| Sleep Apnea | 16/175 (9.1) | 11/249 (4.4) | 2.1732 | 0.0677 |
| Seizure Disorder | 24/175 (13.7) | 32/249 (12.9) | 1.0776 | 0.8843 |

**Supplementary Table 1:** Characteristics and outcomes for NSC and trauma patients. Patient demographics revealed that NSC (n = 175) patients were more likely to be male than the trauma control (n = 249). A significantly higher proportion of NSC patients presented with ophthalmological diagnoses, chronic otitis media, developmental delays, and hearing loss compared to patients in the trauma control group, whereas trauma patients were more likely to present with chronic headaches than NSC patients.

| **Supplementary Table 2. Outcomes and primary NSC diagnosis** | | | | | |
| --- | --- | --- | --- | --- | --- |
| **Outcome** | **Coronal** | **Metopic** | **Pan^a^** | **Sagittal** | **P Value^b^** |
| Chronic Headache | 1/25 (4.0) | 6/48 (12.5) | 1/4 (25.0) | 13/98 (13.3) | 0.4633 |
| Developmental Delay | 10/25 (40.0) | 17/48 (35.4) | 4/4 (100.0) | 28/98 (28.6) | 0.5047 |
| Chronic Otitis Media | 5/25 (20.0) | 14/48 (29.2) | 2/4 (50.0) | 21/98 (21.4) | 0.5542 |
| Ophthalmological Diagnoses | 6/25 (24.0) | 17/48 (35.4) | 3/4 (75.0) | 32/98 (32.7) | 0.5822 |
| Seizure Disorder | 5/25 (20.0) | 7/48 (14.6) | 0/4 (0.0) | 12/98 (12.2) | 0.6592 |
| Hearing Loss | 3/25 (12.0) | 6/48 (12.5) | 2/4 (50.0) | 8/98 (8.2) | 0.7066 |
| Sleep Apnea | 1/25 (4.0) | 4/48 (8.3) | 3/4 (75.0) | 8/98 (8.2) | 0.8441 |
| ^a^ Not used in P Value calculation due to inadequate sample size | | | | | |
| ^b^ P Value computed using the χ^2^ Test  **Supplementary Table 2:** Outcomes and primary NSC diagnosis. No significant associations were found between any of the observed outcomes and NSC subtype diagnosis. | | | | | |

| **Supplementary Table 3. Outcomes of male and female NSC patients using odds ratios** | | | | |
| --- | --- | --- | --- | --- |
| **Outcome** | **Female** | **Male** | **Odds Ratio** | **P Value** |
| Ophthalmologic Diagnoses | 32/66 (48.5) | 26/109 (23.9) | 2.9842 | 0.0010 |
| Hearing Loss | 13/66 (19.7) | 6/109 (5.5) | 4.1743 | 0.0052 |
| Sleep Apnea | 10/66 (15.2) | 6/109 (5.5) | 3.0447 | 0.0549 |
| Seizure Disorder | 6/66 (9.1) | 18/109 (16.5) | 0.5074 | 0.1830 |
| Developmental Delay | 25/66 (37.9) | 34/109 (31.2) | 1.3427 | 0.4108 |
| Chronic Headache | 9/66 (13.6) | 12/109 (11.0) | 1.2745 | 0.6362 |
| Chronic Otitis Media | 16/66 (24.2) | 26/109 (23.9) | 1.0214 | 1.0000 |

**Supplementary Table 3:** Outcomes for male and female NSC patients. A significantly higher proportion of female (n = 66) NSC patients presented with ophthalmological diagnoses and hearing loss compared to male (n = 109) NSC patients.

| **Supplementary Table 4.** **Subgroup analyses for the association of sex and ophthalmologic diagnoses using odds ratios** | | | | | |
| --- | --- | --- | --- | --- | --- |
| **Age (months)** | **Outcome** | **Female** | **Male** | **Odds Ratio** | **P Value** |
| All Data | Ophthalmologic Diagnoses | 32/66 (48.5) | 26/109 (23.9) | 0.3351 | 0.0010 |
| ≤6 | Ophthalmologic Diagnoses | 8/23 (34.8) | 8/42 (19.0) | 0.4472 | 0.2287 |
| 6 - 12 | Ophthalmologic Diagnoses | 11/21 (52.4) | 8/27 (29.6) | 0.4292 | 0.1426 |
| 12-24 | Ophthalmologic Diagnoses | 9/14 (64.3) | 5/17 (29.4) | 0.1973 | 0.0759 |
| ≥ 24 | Ophthalmologic Diagnoses | 4/8 (50.0) | 5/23 (21.7) | 0.5680 | 0.1845 |

**Supplementary Table 4:** Subgroup analyses for the association of sex and ophthalmological diagnoses among NSC patients. Female NSC patients (n = 66) consistently demonstrated higher rates of presentation with ophthalmological diagnoses than male NSC patients (n = 109).

| **Supplementary Table 5.** **Subgroup analyses for the association of sex and hearing loss using odds ratios** | | | | | |
| --- | --- | --- | --- | --- | --- |
| **Age (months)** | **Outcome** | **Female** | **Male** | **Odds Ratio** | **P Value** |
| All Data | Hearing Loss | 13/66 (19.7) | 6/109 (5.5) | 0.2369 | 0.0052 |
| ≤ 6 | Hearing Loss | 2/23 (8.7) | 3/42 (7.1) | 0.8104 | 1.0000 |
| 6 - 12 | Hearing Loss | 6/21 (28.6) | 2/27 (7.4) | 0.2070 | 0.1146 |
| 12 - 24 | Hearing Loss | 3/14 (21.4) | 0/17 (0.0) | 0.0000 | 0.0810 |
| ≥ 24 | Hearing Loss | 2/8 (25.0) | 1/23 (4.3) | 0.1482 | 0.1557 |

**Supplementary Table 5:** Subgroup analyses for the association of sex and hearing loss among NSC patients. Female NSC patients (n = 66) consistently demonstrated higher rates of presentation with hearing loss than male NSC patients (n = 109).

| **Supplementary Table 6.** **Outcomes for male and female trauma patients using odds ratios** | | | | |
| --- | --- | --- | --- | --- |
| **Outcome** | **Female** | **Male** | **Odds Ratio** | **P Value** |
| Seizure Disorder | 21/124 (16.9) | 11/125 (8.8) | 0.4747 | 0.0605 |
| Ophthalmologic Diagnoses | 8/124 (6.5) | 17/125 (13.6) | 2.2751 | 0.0901 |
| Developmental Delay | 16/124 (12.9) | 11/125 (8.8) | 0.6524 | 0.3160 |
| Chronic Otitis Media | 4/124 (3.2) | 2/125 (1.6) | 0.4892 | 0.4465 |
| Hearing Loss | 3/124 (2.4) | 6/125 (4.8) | 2.0280 | 0.4998 |
| Chronic Headache | 22/124 (17.7) | 27/125 (21.6) | 1.2761 | 0.5242 |
| Sleep Apnea | 4/124 (3.2) | 7/125 (5.6) | 1.7756 | 0.5395 |

**Supplementary Table 6:** Outcomes for male and female trauma patients. No significant associations were found between any of the observed outcomes and male (n = 125) or female (n = 124) trauma patients.
